# ColoWeb: a resource for analysis of colocalization of genomic features

**DOI:** 10.1186/s12864-015-1345-3

**Published:** 2015-02-28

**Authors:** RyangGuk Kim, Owen K Smith, Wing Chung Wong, Alex M Ryan, Michael C Ryan, Mirit I Aladjem

**Affiliations:** DNA Replication Group, Laboratory of Molecular Pharmacology, Developmental Therapeutics Branch, National Cancer Institute, National Institutes of Health, Bethesda, Maryland USA; In Silico Solutions, Falls Church, VA USA

**Keywords:** ChIP-seq, Epigenetics, Genomics, Sequencing, Web service

## Abstract

**Background:**

Next-generation sequencing techniques such as ChIP-seq allow researchers to investigate the genomic position of nuclear components and events. These experiments provide researchers with thousands of regions of interest to probe in order to identify biological relevance. As the cost of sequencing decreases and its robustness increases, more and more researchers turn to genome-wide studies to better understand the genomic elements they are studying. One way to interpret the output of sequencing studies is to investigate how the element of interest localizes in relationship to genome annotations and the binding of other nuclear components. Colocalization of genomic features could indicate cooperation and provide evidence for more detailed investigations. Although there are several existing tools for visualizing and analyzing colocalization, either they are difficult to use for experimental researchers, not well maintained, or without measurements for colocalization strength. Here we describe an online tool, ColoWeb, designed to allow experimentalists to compare their datasets to existing genomic features in order to generate hypotheses about biological interactions easily and quickly.

**Results:**

ColoWeb is a web-based service for evaluating the colocation of genomic features. Users submit genomic regions of interest, for example, a set of locations from a ChIP-seq analysis. ColoWeb compares the submitted regions of interest to the location of other genomic features such as transcription factors and chromatin modifiers. To facilitate comparisons among various genomic features, the output consists of both graphical representations and quantitative measures of the degree of colocalization between user’s genomic regions and selected features. Frequent colocation may indicate a biological relationship.

**Conclusion:**

ColoWeb is a biologist-friendly web service that can quickly provide an assessment of thousands of genomic regions to identify colocated genomic features. ColoWeb is freely available at: http://projects.insilico.us.com/ColoWeb.

**Electronic supplementary material:**

The online version of this article (doi:10.1186/s12864-015-1345-3) contains supplementary material, which is available to authorized users.

## Background

Functional analyses of genomic features, such as chromatin immunoprecipitation followed by massive parallel sequencing (ChIP-seq) [1,2], have quickly been recognized as useful tools for examining the components that modulate chromatin. The integration of epigenetic information with genome sequences is essential for a complete understanding of genome organization and function [3]. Massive parallel sequencing allows researchers to identify DNA sequences associated with particular chromatin functions such as protein binding, DNA replication initiation or transcriptional activation. These experiments reveal where distinct chromatin modifications occur and provide clues to the function of protein-DNA interactions.

Several combined community efforts have amassed datasets of chromatin features that are publically available and can be used to show the relationship between certain nuclear elements and functional output [2,4,5]. These datasets help to categorize genomic regions and assist in the deconvolution of the relationships between genetic sequences and complex human biology. The many different cell-types and differentiation states that can arise from a single human genome emphasize the importance of epigenetic regulation that controls biological outcome.

One technique to understand the role that nuclear elements play in controlling genome function and organization is to compare the locations of genomic regions involved in particular interactions with the locations other known elements. Tools that perform analyses of this kind are available on software suites and within bioinformatic pipeline packages [6,7]. Some tools can compare user-provided lists of regions, but are limited to general genome annotations (e.g. transcriptional start sites (TSS) and CpG islands (CGI)) [8,9]. Others are web-based, and allow users to compare input files to available chromatin features, but lack a graphical output that provides a continuous representation of colocalization over a sliding window [10]. Further, these tools provide one-way analyses and do not allow for reciprocal comparisons. The tool described below, ColoWeb, is a web-based tool that allows researchers to submit files containing genomic regions and then quantifies the extent of association between the provided regions and previously identified genome-wide features.

## Implementation

ColoWeb is a web-based application that accepts a region of interest (ROI) file from users in .bed format. Generally ROI files contain thousands of genomic locations of a particular biological event produced from a peak selection program. The submitted ROIs are compared to a cell line-specific library of genomic features obtained from the UCSC Genome Browser [2]. If the pre-built features are not appropriate for a particular study, users may upload a genomic feature file in .bed format along with their ROI file. For each genomic feature, colocation analysis is performed to identify how often and in what spatial pattern the feature is in proximity to the user submitted ROIs. ColoWeb produces a feature density image, histogram, and several summary statistics for each feature that quantify colocalization.

### Analysis

**Figure 1:**
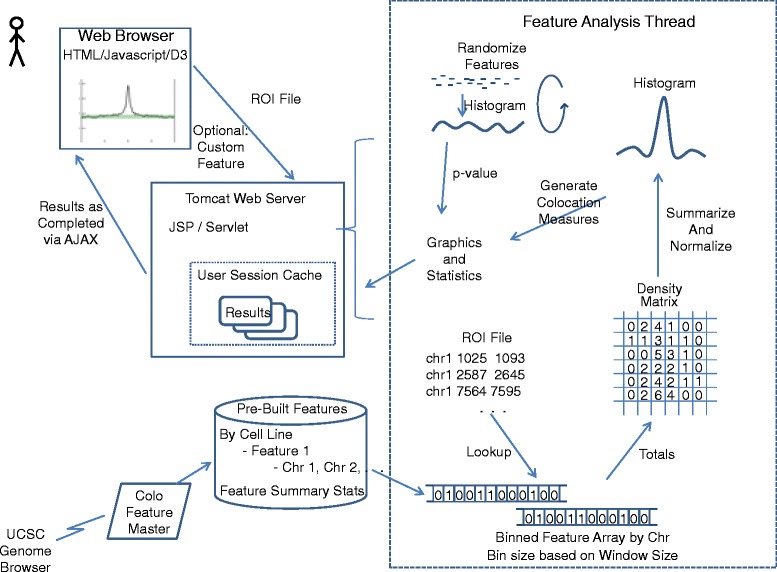
**Architecture and analysis flow of ColoWeb.** A pre-built library of cell line specific genomic features split by chromosome is automatically built using data pulled from the UCSC Genome browser. Users submit a region of interest file via their browser to our Tomcat server. An analysis thread is started for each user request. The thread analyses a series of genomic features one at a time. Binned feature arrays tuned to the size of the analysis window are used to efficiently construct a density matrix of feature totals relative to the center of submitted regions (center matrix column). Column totals from the density matrix are normalized and summarized into a histogram. Colocation statistics including % of regions colocalized, peak height, AMI, and BMI are calculated. An empirical p_value for the AMI is calculated by repeated random permutation of features. Graphical and quantitative results for each genomic feature are placed in a user specific session cache and returned to the user via AJAX as analysis of each feature completes.

ColoWeb is designed for rapid genome-wide analysis of many genomic features. A pre-built library of genomic features is created by an automated process that downloads features from UCSC Genome Browser, splits features into a file per chromosome, and generates summary statistics for the feature (number of features, average width, etc.). Each pre-built per chromosome feature can be quickly loaded into a binned array in memory to support rapid lookup of any location in the chromosome and identify features within a given window size of the lookup location. When a user submits a ROI file, the following analysis steps are performed for each genomic feature analyzed (Figure 1):

1. A feature density matrix is created. Each row of the matrix contains 100 bins. The number of base pairs in each bin is determined by the user configurable window size. If the user selects a 20,000 bp window, then each bin will cover 200 bases. The matrix has 100 rows, each summarizing 1/100^th^ of the user submitted ROIs. For example, if the user submits 5000 regions, then each row will summarize 50 ROIs.
2. From the center of each user submitted region, a lookup is performed to find bins within the analysis window size that contain the genomic feature. The center of a matrix row represents the center of the user ROI. The total in the appropriate bins are incremented when a genomic feature is found in the bin position relative to the user ROI. The generated density matrix is present to user as an image. The shading of the image is scaled based on the highest bin total in the image with dark areas indicating a high density of features.
3. A histogram is created by summing the columns of the density matrix into a single line chart of the total genomic features observed relative to the center of the user ROIs for the selected window size. To facilitate cross-feature and cross-sample analysis, feature totals are normalized by the number of user regions (totals are per 10,000 user regions) and by the number and size of the genomic feature (totals are per 10,000 feature bins – the total number of bins that the feature occupies in the genome).
4. A variety of basic statistics are calculated for each feature: number of genomic features, average feature size, and percentage of user peaks with at least one co-located feature.
5. To quantify the strength of colocalization we calculate a colocation peak height, Above-Mean-Integral (AMI), and Below-Mean-Integral (BMI) from the histogram. First, we estimate the background level of feature density (*L*_*b*_) by taking the average value from the histogram bins (*v*_*b*_) that are furthest from the ROI. Bins up to and including the 25^th^ bin (*Q*_1_) and those from the 75^th^ (*Q*_3_) and higher are used.

$$ {S}_b=\left\{{v}_b\Big|{v}_b\le {Q}_1 and\ {v}_b\ge {Q}_3\right\} $$

$$ {L}_b=\frac{{\displaystyle {\sum}_{v_b\in {S}_b}}{v}_b}{\left|{S}_b\right|} $$

Next, we estimate the background variance in feature density (*V*) using the 75th percentile oscillation distance from an ordered list of increase / decrease distances observed when traversing the histogram. The estimated background and upper/lower variance from background are drawn as green lines on the histogram. The colocation peak height (H) is maximum histogram value above the upper variance level. AMI is the integral of histogram values above the upper variance level. BMI is calculated to quantify anti colocation and is the integral of histogram values below the lower variance level.

$$ {L}_h={L}_b+V $$

$$ {L}_l=V-{L}_b $$

$$ {S}_h=\left\{{v}_b\Big|{v}_b>{L}_h\right\} $$

$$ {S}_l=\left\{{v}_b\Big|{v}_b<{L}_l\right\} $$

$$ H=\underset{v_b\in {S}_h}{ \max }{v}_b $$

$$ AMI={\displaystyle \sum_{v_b\in {S}_h}}{v}_b $$

$$ BMI={\displaystyle \sum_{v_b\in {S}_l}}{v}_b $$

ColoWeb calculates a conservative, empirical p-value by randomizing features within the histogram. The AMI is calculated for histograms with randomized feature placement. The randomization / AMI calculation is performed repeatedly and the percentage of randomized histograms with AMIs > = the actual histogram is returned as the p-value.

If a user provides a custom genomic feature, it is run through the same steps as our pre-built features to split it by chromosome and calculate summary feature statistics. Processing is then identical for pre-built and user submitted genomic features. If a user selects feature-centered analysis, the processing described above is inverted so that the density matrix represents each genomic feature at its center and the position of user regions is displayed as a density plot and histogram. Aside from inverting the region of interest and genomic features, processing is identical.

### Technical architecture

ColoWeb is implemented as a Java servlet running on a Tomcat server, and its user interface is written in JSP/Javascript with the help of the libraries JQuery, JTable and JSZip. User submitted data is received by a servlet and processed in a separate analysis thread that has access to our pre-built library of genomic features and feature statistics. Results are presented to the user incrementally via AJAX as analysis of each feature completes, with the density image and histograms rendered using the D3 and CanVG Javascript libraries. After the last feature is analyzed, the histograms are dynamically re-scaled in the browser to the highest feature total, while the rows are resorted by descending AMI.

### Usage

ColoWeb is intended for analysis of relatively fine-grained genomic features less than a few thousand bases wide. A simple tab delimited text file (.bed format) containing the genomic position of user regions of interest is submitted to ColoWeb. Only the first three fields of .bed format are required: chromosome, start position, and stop position. The upper limit of user feature width is ¼ of the window size selected for analysis (e.g. 5,000 bases for a 20,000 base window). Regions larger than the limit will be discarded and there must be at least 1000 valid regions to perform colocation analysis. For performance reasons, we process only up to 200,000 submitted user regions. If more are submitted, ColoWeb will randomly sample 200,000 for colocation analysis.

The cell line in which the user regions were identified should be selected for colocation analysis. If ColoWeb does not have pre-built features for the cell line used, select the ‘Any Human’ features, upload a custom feature file, or contact us to add cell lines / features. The current version supports analyses for only the human genome.

Specification of window size provides a mechanism to zoom in or out for tighter or wider colocation analysis. The drawback of larger window sizes is that they are more likely to overlap with other ROIs, confounding the colocation analysis. Generally the most intuitive analysis is centered on the submitted ROI. Occasionally the view presented by feature-centered analysis provides additional insights. ColoWeb makes it easy to try out different feature sets, window sizes, and analysis options.

A full set of results including images can be saved with the ‘Save’ button on the results screen, which will deliver a .zip file. The quantitative data in a form more easily imported to a spreadsheet can be obtained with the ‘Data’ button on the results page.

## Results

ColoWeb enables researchers to quickly and easily explore the genomic features that are co-located with regions they are studying. Users provide a bed file with their regions of interest and select the feature set they would like to analyse. Results are generally provided in less than a minute. The results can be explored and dynamically sorted in the browser and exported as a zip file with images and data or just a text file of the data values.

Three practical examples are used to demonstrate the ColoWeb tool. First a recently published dataset of regions bound by a transcriptional regulator are compared to general genomic features and those specific to the cell line of origin, K562. Second, due to enrichment of shared features this transcriptional regulator is compared to K562 replication origins, a user uploaded file. ColoWeb assists in identifying one chromatin feature that distinguishes between the transcriptional regulator and replication origins. For the third example, early and late firing replication origins are compared using the quantification of colocalization with epigenetic features generated by ColoWeb. Coloweb identifies features that colocalize with all replication origins and features found to be associated with early, but not late firing replication origins.

In the first example, we analyzed epigenetic features colocalizing with a recently published dataset of the TFII-I human transcription factor binding sites [11]. Binding sites were identified by a ChIP-seq experiment with an epitope-tagged species of this transcriptional regulator stably expressed in K562 erythroleukemia cells. The original analysis suggests the TFII-I is bound to both activated and repressed genes and associated with both activating and repressive histone marks [11]. We compared TFII-I binding sites to the common features of all cell lines and to the list of features for K562 defined as “modifiers” using ColoWeb. A bed file containing 10,359 enriched TFII-I regions aligned to the human genome (hg19) was downloaded from GEO (http://www.ncbi.nlm.nih.gov/geo/query/acc.cgi?acc=GSE51065) and input to ColoWeb**.**

**Figure 2:**
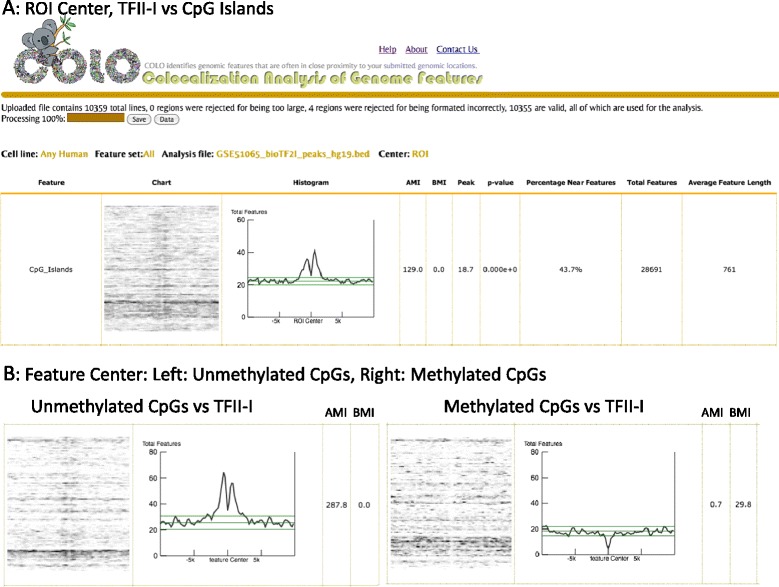
**Colocation analysis of TFII-I bound regions with pre-computed ColoWeb features.** Select output of the comparison of TFII-I bound regions to **A)** the Any Human feature set (CpG islands) centered on TFII-I and with a 20 kb window size and **B)** the K562 Modifiers set, centered on TFII-I and with a 20 kb window size. Left: Unmethylated CpGs, Right: methylated CpGs.

To obtain a more general sense of TFII-I binding, this file was compared to features common among all cell lines with the plot centered on TFII-I enriched regions. ColoWeb identified CpG islands as being significantly enriched for TFII-I binding (Figure 2A). CGIs accumulated both upstream and downstream of TFII-I binding, but not in the middle of TFII-I bound loci. We then repeated the analysis, this time centering the colocalization on the feature and overlaying TFII-I binding. A similar pattern was found indicating a shared exclusion of TFII-I from CGIs as well as a strong accumulation of TFII-I both upstream and downstream of CGIs (Additional file 1: Figure S1A).

Since methylation is cell-specific, we used ColoWeb to compare TFII-I binding sites to a list of K562 modifiers that includes regions of both methylated and unmethylated CpGs. As shown in Figure 2B, TFII-I binding sites showed a strikingly high enrichment to unmethylated CpGs, while still displaying the bifurcated distribution. TFII-I exhibited no colocalization with methylated CGIs. The absence of TFII-I binding from methylated CGIs may be related to the well-established observation that DNA hypermethylation is associated with gene inactivation, potentially by blocking binding of transcription factors to DNA [12,13]. Again we have observed a similar relationship when the analysis was centered on TFII-I binding regions. (Additional file 1: Figure S1B). In addition, ColoWeb confirms the observed association between TFII-I and E2Fs (Additional file 2: Figure S2) noted by Fan *et al.* [11].

**Figure 3:**
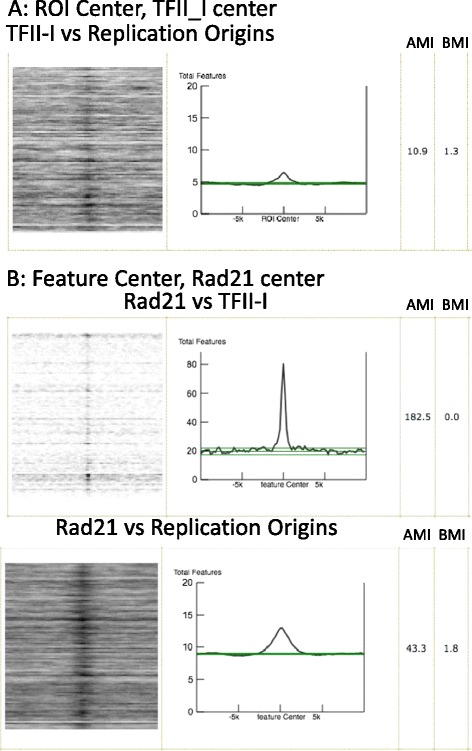
**Colocation analysis of TFII-I bound regions with a user supplied genomic feature. A)** Comparison of TFII-I bound regions to another user-provided file, K562 replication origins, centered on TFII-I, considering a 20 kb window size, **B)** comparison of TFII-I bound regions and K562 replication origins to Rad21 (from K562 Modifiers set), with the analysis centered on Rad21, considering a 20 kb window size.

Similar to the association of TFII-I transcriptional regulator, initiation sites of DNA replication (i.e. replication origins) have also been shown to associate predominantly with CGIs [14,15] and with transcription factor binding sites [16-18]. To explore whether the shared association with CGIs suggests a potential role for TFII-I binding in DNA replication, we investigated the extent of colocalization of TFII-I binding sites with replication origins, utilizing the capability of ColoWeb to compare two user-provided files (Figure 3A). A moderate colocalization was observed with the analysis centered on TFII-I, suggesting that TFII-I does not have a major role in the regulation of DNA replication. A reciprocal colocalization showed almost no colocalization of TFII-I bound regions to replication origins (Additional file 3: Figure S3A). In addition, both TFII-I binding sites and replication origins were compared to K562 modifiers. We observed a discrete colocalization of Rad21 with TFII-I and not with DNA replication initiation (Figure 3B for feature-centered analysis, compare AMIs, 182.5 for TFII-I to 43.3 for replication origins, and Additional file 3: Figure S3B for region-centered analysis). Both K562 replication origins and TFII-I binding regions have strong associations with CpG islands, but these data sets are clearly distinguished by their relationship to Rad21.

**Figure 4:**
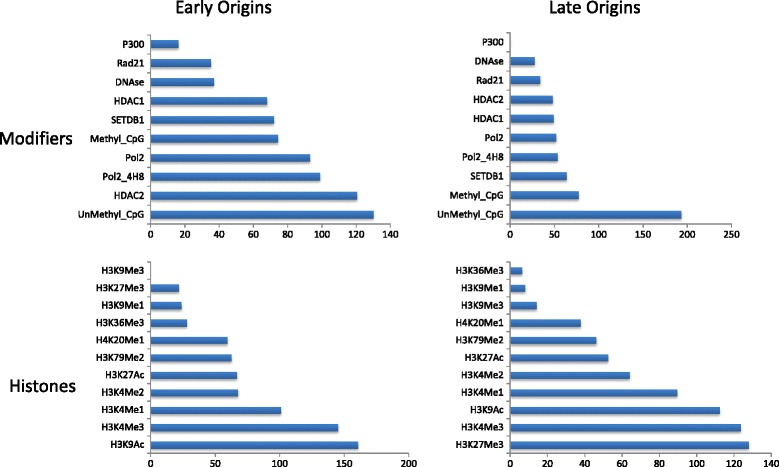
**Histogram of AMI Values.** AMI values output from ColoWeb when early and late replication origins from Erythroblast cells are compared to K562 modifiers and histones.

Coloweb allows users to download the generated AMIs in a csv file that can easily be used for direct comparison between features. In the third example that illustrates the advantage of ColoWeb’s quantification, a recently published data set of erythroblast cell origins, and their respective replication timing, was used to generate two files, one composed of the early replicating origins and another composed of the late replicating origins [19]. These erythroblast replication origins were compared to K562 features, an erythroid cell line available on ColoWeb. After early and late origins were compared to K562 “modifiers” and “histones”, the AMI values were exported, and histograms were created to investigate colocalization between these two sets of replication origins (Figure 4). Some features appear to be associated with both early and late replication origins (Unmethylated CpGs, H3K4me3, H3K9Ac). In contrast, colocalization with HDAC2 is high in the early origins and low in the late origins. This comparison of replication origins and epigenetic features was easy to perform using the ColoWeb tool, provides output comparable to previously published work (See [14] figure three), and can lead to the generation and rapid testing of meaningful hypotheses.

## Discussion

There are other good tools that quantify colocalization of genomic regions [6-10] and these software packages provide a variety of capabilities and analysis strengths (Additional file 4: Figure S4, Additional file 5: Table S1). In our lab, we found that the existing tools did not meet the needs of the bench biologists to quickly and easily screen new ChIP-seq data for colocation with many existing datasets of genomic features. We needed results that were both graphical and numerical quantifications and that would enable cross-feature, cross-sample, and cross-cell line comparisons. Further, we decided that a web-based service and a pre-built library of genomic features would best support researchers without requiring software installation or bioinformatics support.

One significant advantage of ColoWeb is its numerical measures of the strength of colocalization, AMI. Among the other publicly available colocation tools of which we are aware, only seqMiner provides a colocalization strength measure (ChIP-seq enrichments, defined as log2[(reads in the window from the data ROIs + q)/(reads in the window from the control ROIs + q)] where q is about 10). The seqMiner approach has difficulty with sharp and narrow peaks from features with high background read levels because the peak’s size can be small relative to background reads from the control. This type of peak can, however, indicate a very focused and interesting colocalization. ColoWeb’s AMI instead uses the edges of the histogram itself and observed histogram variance to establish a dynamic background level for each feature. We find that this approach is able to accurately quantify a wide range of colocation patterns in histograms. Further, our ROI and feature bin normalization combined with dynamic background estimation allows the ColoWeb AMIs to be directly compared for cross-feature or cross-sample assessment.

ColoWeb easily and quickly generates meaningful colocalization outputs that can assist users in forming initial impressions and in validating their sequencing results. We have provided three examples for hypothesis generation and testing using Coloweb. In the first example, a recently published dataset was subjected to analysis using K562 features, centering on both the feature and TFII-I allowing for the generation of the novel hypothesis that TFII-I binds to unmethylated but not methylated CGIs. When performing the reciprocal analysis, centering on the feature, it is not always expected that the colocalization will be similar. In particular, if the file has few peaks, reciprocal overlap may be less likely because fewer genomic regions are considered. In this instance the ROI-centered and feature-center plots are similar.

In the second example, we surveyed the colocalization of chromatin components with replication origins to evaluate whether origins and TFII-I binding sites share common associations. This analysis revealed that although TFII-I binding sites and origins associate with many common features, they differ in their association with Rad21. Rad21 is a member of the cohesin complex, which remains bound to chromatin throughout the cell cycle and is involved in maintaining higher order chromatin structure to allow for competency of both transcription and DNA replication [20,21]. Rad21 shows a strong colocalization with TFII-I binding sites, with a very discrete relationship supporting shared function, while Rad21 only had modest colocalization with K562 replication origins. Therefore TFII-I may be cooperating with the cohesin complex to promote its transcriptional program, particularly in the presence of CGIs, and in a manner distinct from the regulation of DNA replication.

The final example demonstrates how ColoWeb can be used to compare the epigenetic signature of two files. ColoWeb was used to characterize early and late replicating origins with the goal of identifying chromatin features that may be involved in regulating replication timing. The AMI value that quantifies the degree of colocalization was used to compare several features to both early and late firing replication origins. Histograms of K562 “histones” and “modifiers” displayed that several epigenetic features are associated with both early and late firing origins. HDAC2 was identified as having a high AMI value for early firing replication origins, but a low AMI for late those that are late firing. This finding suggests that HDAC2 may be involved in the regulation of replication timing.

The quantitative output that measures the degree of colocalization is unique in ColoWeb and of a potentially great value to users. Bed file intersections can provide the percentage of features within a certain distance from a reference file, which ColoWeb provides in the “Percentage Near Feature” output, but ColoWeb also outputs a normalized quantification of the degree of positive colocalization, AMI, or of the absence of colocalization, BMI. These values are suitable for direct comparison between features, which is difficult with graphical outputs provided by other tools, as shown in the comparison of early and late replication origins using ColoWeb. Additionally the Coloweb tool outputs a p-value for the AMI that provides users with confidence in the significance of the colocalization. The flexibility and ease of use found in the ColoWeb tool presents advancement from existing tools and will greatly increase the ability of any scientist to analyse and compare results from sequencing experiments.

## Conclusions

The ColoWeb tool is a rapid web-based service that is readily available to the public and allows for bed file comparisons between user-provided input and many publically available features. ColoWeb has stored several features from separate cell lines to allow users to make cell-line specific comparisons, as well as general features common to all cell lines. This tool has an easy to use interface that can center its analysis on stored features or user-provided regions of interest, and can consider flexible distance from reference regions. ColoWeb outputs quantification of colocalization and statistical analysis, which are not available in current tools. This tool allows researchers to quickly and easily compare several features without great difficulty, providing both those gaining experience with bioinformatics and experienced bioinformaticians with an easy platform that can inform more detailed investigations.

## Availability and requirements

**Project name**: **ColoWeb**

**Project home page**: **http://projects.insilico.us.com/ColoWeb**

**Operating system**: Platform independent.

**Programming language**: Java, Javascript, JSP.

**Other requirements**: none.

**License**: Free to use.

**Any restrictions to use by non-academics**: **None.**

## Additional files

Additional file 1: Figure S1. Select output of the comparison of TFII-I bound regions to A) the Any Human feature set (CpG islands) centered on CpG islands and with a 20 kb window size and B) the K562 Modifiers set, with a 20 kb window size, centered on: Unmethylated CpGs (left), methylated CpGs (right). Additional file 2: Figure S2. Select output (E2Fs) of the comparison of TFII-I bound regions to the K562 Modifiers feature set, centered on the user-provided ROI and with a 20 kb window size. Additional file 3: Figure S3. A) Comparison of TFII-I bound regions to another user-provided file, K562 replication origins, centered on replication origins, considering a 20 kb window size, B) comparison of TFII-I bound regions and K562 replication origins to Rad21 (from K562 Modifiers set), with the analysis centered on TFII-I (top) and replication origins (bottom), considering a 20 kb window size. Additional file 4: Figure S4. Example of colocation analysis output from some of the other tools mentioned in **Table S1** for TFII-I bound regions compared to methylated and unmethylated CpG islands. Note EpiGRAPH could not accept hg19 coordinates and could not accept our large CpG island feature set. ngs.plot does not accept .bed files. Output from these tools are not shown. Additional file 5: Table S1. Comparison of the features of ColoWeb with existing tools for analyzing and visualizing sets of genomic locations.

## References

[1] Robertson G, Hirst M, Bainbridge M, Bilenky M, Zhao Y, Zeng T (2007). Genome-wide profiles of STAT1 DNA association using chromatin immunoprecipitation and massively parallel sequencing. Nat Methods.

[2] Karolchik D, Barber GP, Casper J, Clawson H, Cline MS, Diekhans M (2014). The UCSC Genome Browser database: 2014 update. Nucleic Acids Res.

[3] Misteli T (2013). The cell biology of genomes: bringing the double helix to life. Cell.

[4] Consortium EP (2012). An integrated encyclopedia of DNA elements in the human genome. Nature.

[5] Barrett T, Wilhite SE, Ledoux P, Evangelista C, Kim IF, Tomashevsky M (2013). NCBI GEO: archive for functional genomics data sets–update. Nucleic Acids Res.

[6] Quandt K, Grote K, Werner T (1996). GenomeInspector: a new approach to detect correlation patterns of elements on genomic sequences. Comput Appl Biosci.

[7] Quandt K, Grote K, Werner T (1996). GenomeInspector: basic software tools for analysis of spatial correlations between genomic structures within megabase sequences. Genomics.

[8] Shen L, Shao N, Liu X, Nestler E (2014). ngs.plot: Quick mining and visualization of next-generation sequencing data by integrating genomic databases. BMC Genomics.

[9] Ye T, Krebs AR, Choukrallah MA, Keime C, Plewniak F, Davidson I (2011). seqMINER: an integrated ChIP-seq data interpretation platform. Nucleic Acids Res.

[10] Bock C, Halachev K, Buch J, Lengauer T (2009). EpiGRAPH: user-friendly software for statistical analysis and prediction of (epi)genomic data. Genome Biol.

[11] Fan AX, Papadopoulos GL, Hossain MA, Lin IJ, Hu J, Tang TM (2014). Genomic and proteomic analysis of transcription factor TFII-I reveals insight into the response to cellular stress. Nucleic Acids Res.

[12] Bird AP, Wolffe AP (1999). Methylation-induced repression–belts, braces, and chromatin. Cell.

[13] Mutskov V, Felsenfeld G (2004). Silencing of transgene transcription precedes methylation of promoter DNA and histone H3 lysine 9. EMBO J.

[14] Martin MM, Ryan M, Kim R, Zakas AL, Fu H, Lin CM (2011). Genome-wide depletion of replication initiation events in highly transcribed regions. Genome Res.

[15] Picard F, Cadoret JC, Audit B, Arneodo A, Alberti A, Battail C (2014). The spatiotemporal program of DNA replication is associated with specific combinations of chromatin marks in human cells. PLoS Genet.

[16] Cadoret JC, Meisch F, Hassan-Zadeh V, Luyten I, Guillet C, Duret L (2008). Genome-wide studies highlight indirect links between human replication origins and gene regulation. Proc Natl Acad Sci U S A.

[17] Knott SR, Peace JM, Ostrow AZ, Gan Y, Rex AE, Viggiani CJ (2012). Forkhead transcription factors establish origin timing and long-range clustering in S. cerevisiae. Cell.

[18] Zhang Y, Xing Y, Zhang L, Mei Y, Yamamoto K, Mak TW (2012). Regulation of cell cycle progression by forkhead transcription factor FOXO3 through its binding partner DNA replication factor Cdt1. Proc Natl Acad Sci U S A.

[19] Mukhopadhyay R, Lajugie J, Fourel N, Selzer A, Schizas M, Bartholdy B (2014). Allele-specific genome-wide profiling in human primary erythroblasts reveal replication program organization. PLoS Genet.

[20] Smith OK, Aladjem MI. Chromatin structure and replication origins: determinants of chromosome replication and nuclear organization. J Mol Biol. 2014;426(20):3330-41. 10.1016/j.jmb.2014.05.027 PMC4177353 24905010

[21] Yan J, Enge M, Whitington T, Dave K, Liu J, Sur I (2013). Transcription factor binding in human cells occurs in dense clusters formed around cohesin anchor sites. Cell.

