# Supplementary material for: ColoWeb: a resource for analysis of colocalization of genomic features
Source: BMC Genomics. 2015 Feb 28;16(1):142. doi: 10.1186/s12864-015-1345-3 (PMC4364483; doi:10.1186/s12864-015-1345-3)
Supplement: Additional file 1: Figure S1. — Select output of the comparison of TFII-I bound regions to A) the Any Human feature set (CpG islands) centered on CpG islands and with a 20 kb window size and B) the K562 Modifiers set, with a 20 kb window size, centered on: Unmethylated CpGs (left), methylated CpGs (right). [file 12864_2015_1345_MOESM1_ESM.pptx]

## Slide 1
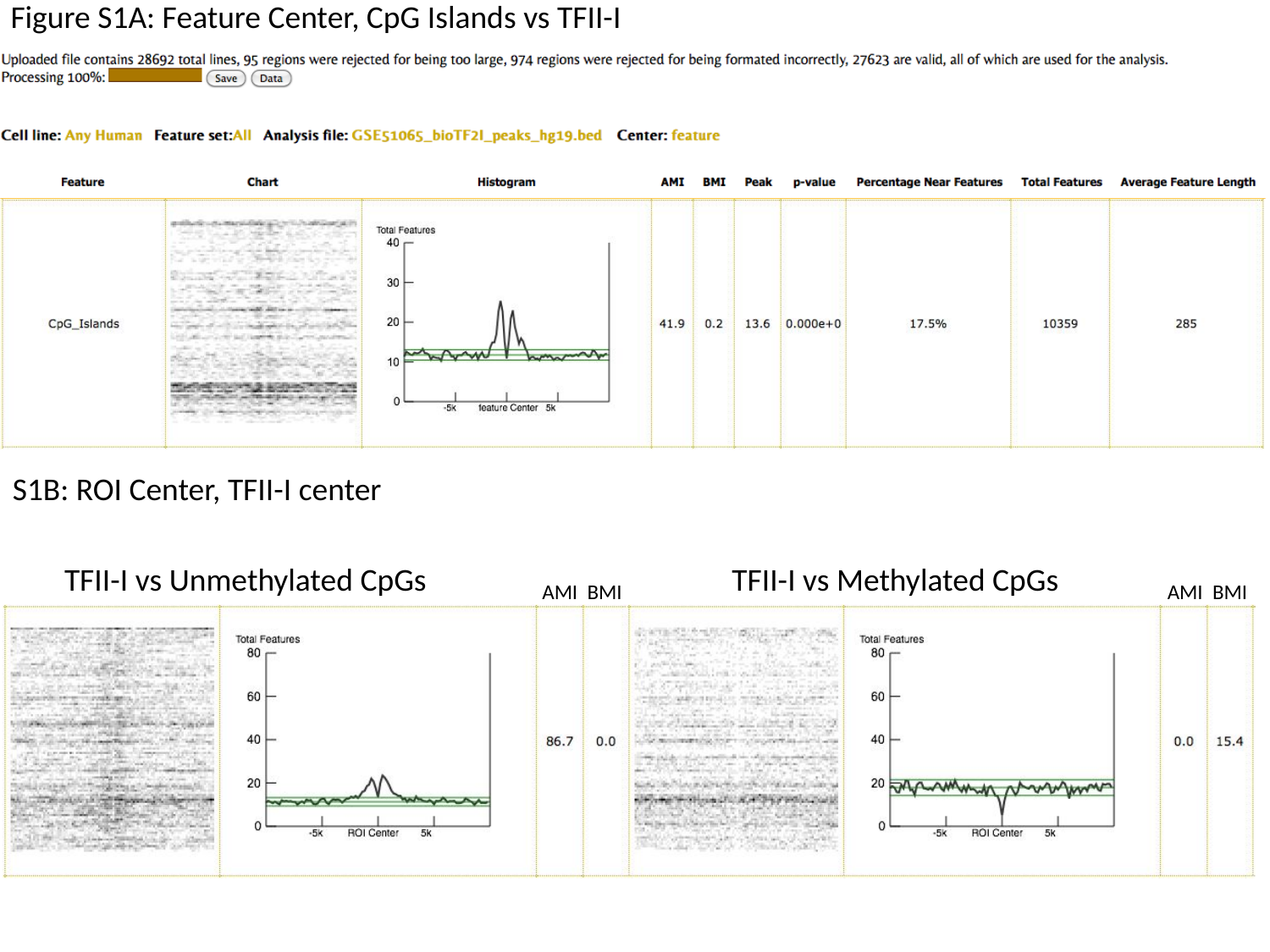

Figure S1A: Feature Center, CpG Islands vs TFII-I
S1B: ROI Center, TFII-I center
TFII-I vs Unmethylated CpGs
TFII-I vs Methylated CpGs
AMI BMI
AMI BMI
