# Supplementary figures and images for: ColoWeb: a resource for analysis of colocalization of genomic features
Source: BMC Genomics. 2015 Feb 28;16(1):142. doi: 10.1186/s12864-015-1345-3 (PMC4364483; doi:10.1186/s12864-015-1345-3)

## Slide 1
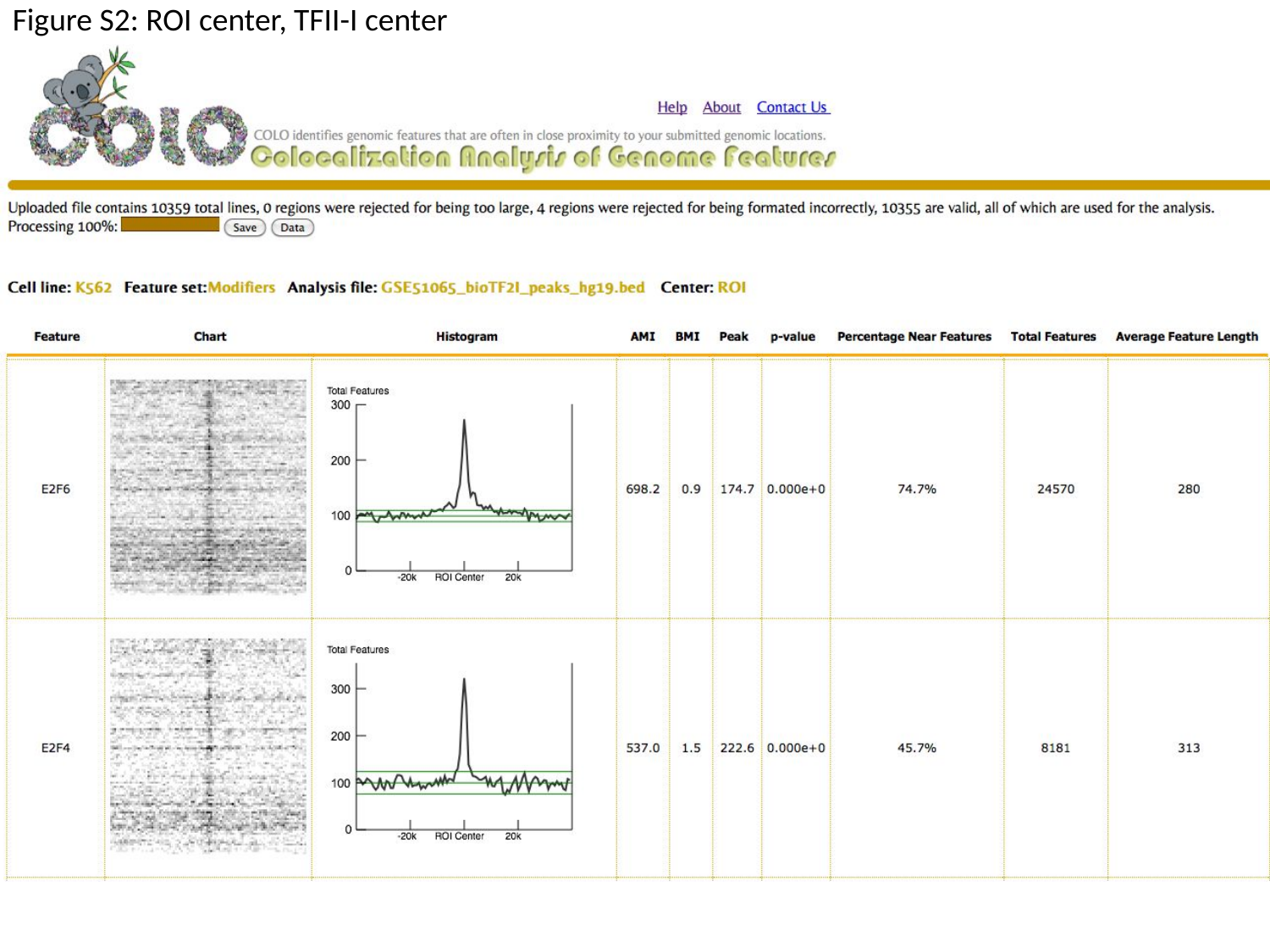

Figure S2: ROI center, TFII-I center

Supplement: Additional file 2: Figure S2. — Select output (E2Fs) of the comparison of TFII-I bound regions to the K562 Modifiers feature set, centered on the user-provided ROI and with a 20 kb window size. [file 12864_2015_1345_MOESM2_ESM.pptx]
