# Supplementary material for: ColoWeb: a resource for analysis of colocalization of genomic features
Source: BMC Genomics. 2015 Feb 28;16(1):142. doi: 10.1186/s12864-015-1345-3 (PMC4364483; doi:10.1186/s12864-015-1345-3)
Supplement: Additional file 3: Figure S3. — A) Comparison of TFII-I bound regions to another user-provided file, K562 replication origins, centered on replication origins, considering a 20 kb window size, B) comparison of TFII-I bound regions and K562 replication origins to Rad21 (from K562 Modifiers set), with the analysis centered on TFII-I (top) and replication origins (bottom), considering a 20 kb window size. [file 12864_2015_1345_MOESM3_ESM.pptx]

## Slide 1
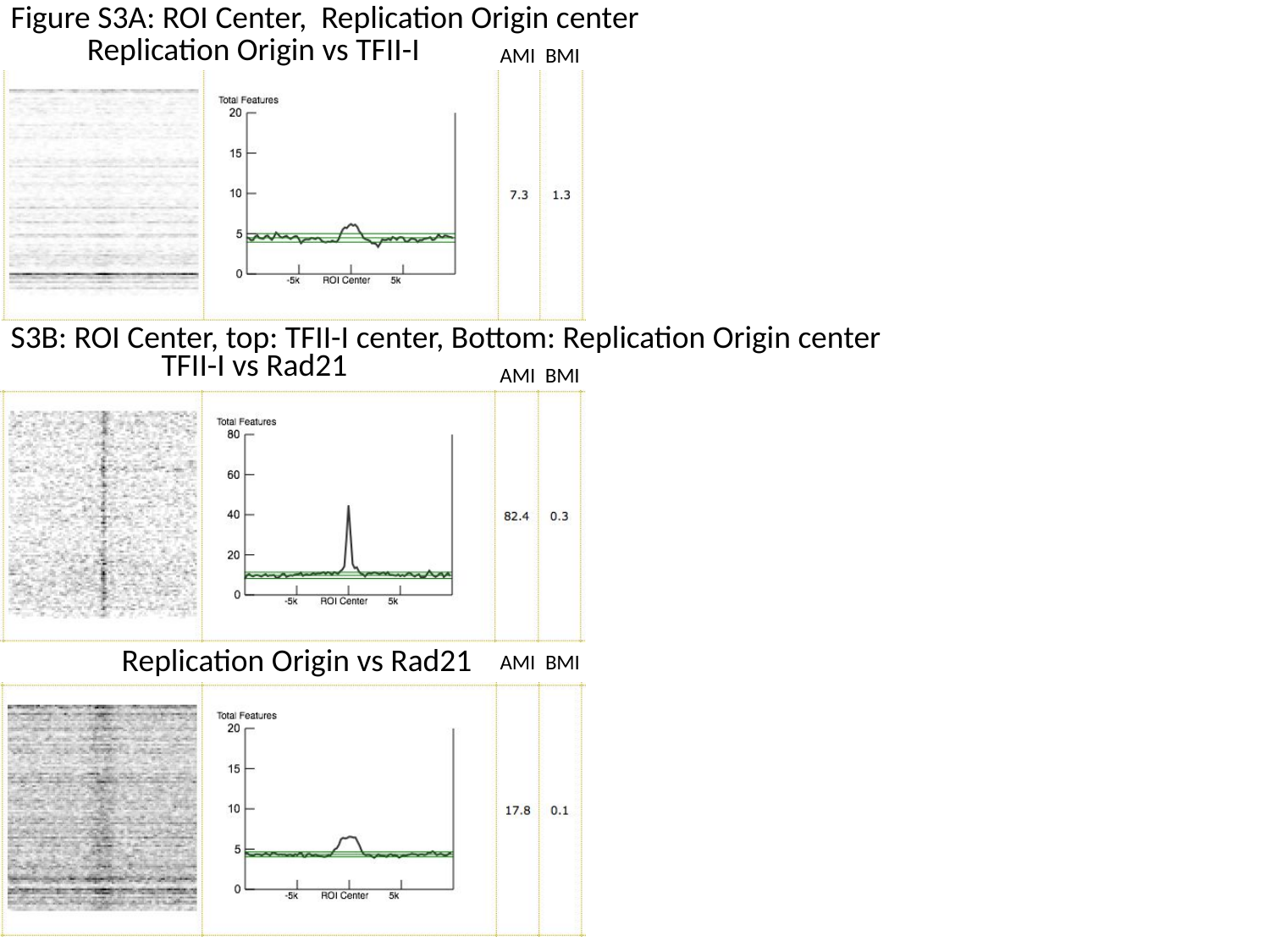

Figure S3A: ROI Center, Replication Origin center
Replication Origin vs TFII-I
AMI BMI
S3B: ROI Center, top: TFII-I center, Bottom: Replication Origin center
TFII-I vs Rad21
AMI BMI
Replication Origin vs Rad21
AMI BMI
