# Supplementary material for: ColoWeb: a resource for analysis of colocalization of genomic features
Source: BMC Genomics. 2015 Feb 28;16(1):142. doi: 10.1186/s12864-015-1345-3 (PMC4364483; doi:10.1186/s12864-015-1345-3)
Supplement: Additional file 4: Figure S4. — Example of colocation analysis output from some of the other tools mentioned in Table S1 for TFII-I bound regions compared to methylated and unmethylated CpG islands. Note EpiGRAPH could not accept hg19 coordinates and could not accept our large CpG island feature set. ngs.plot does not accept .bed files. Output from these tools are not shown. [file 12864_2015_1345_MOESM4_ESM.pptx]

## Slide 1
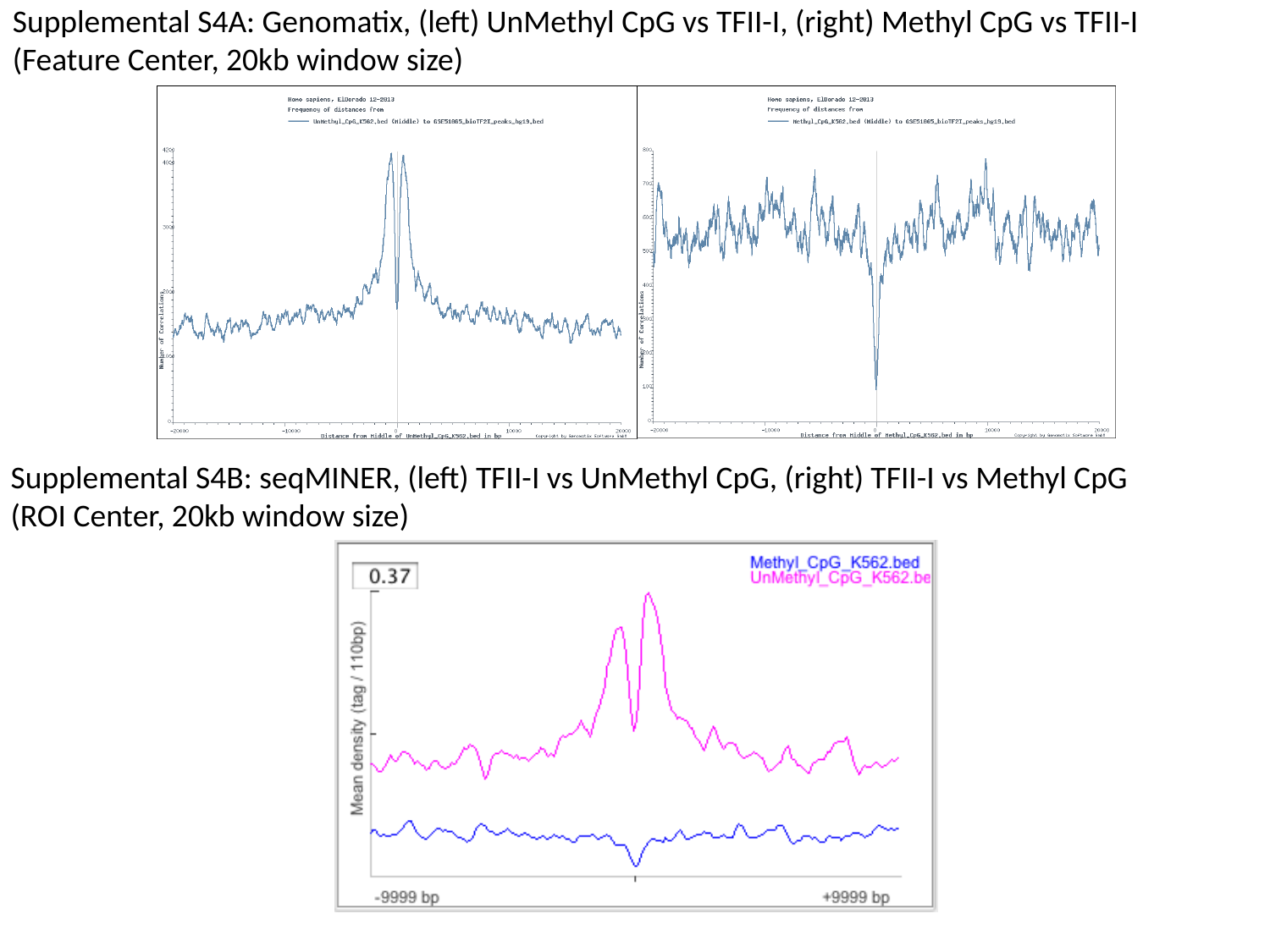

Supplemental S4A: Genomatix, (left) UnMethyl CpG vs TFII-I, (right) Methyl CpG vs TFII-I (Feature Center, 20kb window size)
Supplemental S4B: seqMINER, (left) TFII-I vs UnMethyl CpG, (right) TFII-I vs Methyl CpG (ROI Center, 20kb window size)
